# Editorial: Pattern-recognition receptors: Genetics, immunity, pathology

**DOI:** 10.3389/fcimb.2022.991898

**Published:** 2022-09-20

**Authors:** Petro Starokadomskyy

**Affiliations:** Department of Biophysics, University of Texas (UT) Southwestern Medical Center, Dallas, TX, United States

**Keywords:** interferon, Ifni, pattern recognition receptor (PRR), type I interferonopathy, RIG-I antiviral, cGAS sensor

Innate immunity is the first responder to anything abnormal. One of the branches of innate immunity is type I interferon, or IFNI. Originally, IFNI was discovered as a potent antiviral tool that is employed by all types of cells in response to viral infection (Jensen et al., 1963; Rotem et al., 1963; Liu et al., 2022). Scientists unravel a complex self-regulating network of external and internal receptors, signal kinases, and transcription factors by decoding the molecular mechansms involved in activation of IFNI response. Further biochemical studies reveal the multiple roles of IFNI, including regulation of cell death, inflammation, cell differentiation, transformation and neoplasia, the impact on the level of organism on tissue integrity, vascular and neuronal connectivity, and general health status (McNab et al., 2015). After establishing clinical -omics technologies, disproportional IFNI activation was connected to a number of autoinflammatory disorders, the most prominent of which are grouped into the family of type I interferonopathy (Meyts and Casanova, 2016). Analysis of type I interferonopathy disorders uncovers another role of IFNI signaling – it is deeply involved in the quality control of host RNA. The majority of mutations which activate spontaneous IFNI response belong to the groups of RNA and DNA metabolic enzymes (Starokadomskyy et al., 2021). Mutation and miscommunication between RNA and DNA quality control systems resulted in leakages of toxic RNA/DNA intermediates, which are detecting by Pattern Recognition Receptors (PRRs, including TLR3, RIG-I, cGAS) and mounting a chronic IFNI response leading to pathologies (Farrar, 2014).Therefore, the cell uses the same set of tools for detecting viral infection and mis-spliced or mis-folded self RNA molecules.

In this Research topic, we investigate what else can do IFNI, aiming to analyze non-canonic aspects of the IFNI signaling. For example, human nuclear protein Ku70, involved in DNA non-homologous and joining pathway, also is involved in innate immunity response as DNA sensor, according to the study by Sui et al. This is not the first nuclear DNA enzyme revealing its cytosolic activity as the sensor of foreign nucleic acid. Similarly, POLA1 and other components of DNA polymerase alpha complex (Starokadomskyy et al., 2016), as well as topoisomerase complexes GINSs and MCMs act as a modulator of immune response (Starokadomskyy et al., 2019) (Gineau et al., 2012; Hughes et al., 2012) (Cottineau et al., 2017; Mace et al., 2020).

Another research article from Yang, et al. presents results from studying the potential role of ZAP and TRIM25 proteins in recognition of viral RNA and transcription intervening. Their data suggest that ZAP RNA binding activity acts through specific recognition of CpG dinucleotides in viral RNA. Additionally, RNA binding by ZAP and interaction with TRIM25 may represent two distinct determinants for ZAP antiviral activity in varying viral contexts. This pattern – differential activity which depends on the context, is well illustrated by the review article of the role of IFNI in Falconi anemia pathogenesis (Landelouci et al.). Fanconi Anemia (FA) is a genome instability syndrome caused by mutations in one of the 23 repair genes of the Fanconi pathway. This heterogeneous disease is characterized by congenital abnormalities, premature aging, and bone marrow failure. A defect in one of its proteins prevents functional DNA repair, leading to the accumulation of DNA breaks and genome instability. Abnormal DNA pattern triggers cGAS sensor leading to type I interferon response, exacerbating pathogenesis of the disease (Landelouci et al.). A more systemic view on the interferon pathway and its interaction with cellular metabolism can be found in the review article of Kano et al., summarizing cGAS and other PRRs in developing inflammatory diseases. Innate immunity also can be activated by allergens. In the original research article, Hu et al. showed that Dermatophagoides microceras allergen acts through an LPS-sensing pattern, activating the secretion of universal pro-inflammatory markers IL-6 and IL-8. Given a recent hypothesis connecting allergies to anti-venom immune response (Palm et al., 2012), such interwinding of innate immune signaling and dust mite allergen sensing is intriguing.

Altogether, innate immunity and interferon signaling are versatile tools, developed by cells early in Evolution. Its wide number of cross-connection with the entire metabolism of cells and tissues make it the universal first responder to any problem that cell is facing: from infection to abnormal host metabolism.

## Author contributions

The author confirms being the sole contributor of this work and has approved it for publication.

## Conflict of interest

The author declares that the research was conducted in the absence of any commercial or financial relationships that could be construed as a potential conflict of interest.

## Publisher’s note

All claims expressed in this article are solely those of the authors and do not necessarily represent those of their affiliated organizations, or those of the publisher, the editors and the reviewers. Any product that may be evaluated in this article, or claim that may be made by its manufacturer, is not guaranteed or endorsed by the publisher.
